# TL-118 and Gemcitabine Drug Combination Display Therapeutic Efficacy in a MYCN Amplified Orthotopic Neuroblastoma Murine Model – Evaluation by MRI

**DOI:** 10.1371/journal.pone.0090224

**Published:** 2014-03-06

**Authors:** Chani Komar-Stossel, Eitan Gross, Elia Dery, Nathalie Corchia, Karen Meir, Iris Fried, Rinat Abramovitch

**Affiliations:** 1 The Goldyne Savad Institute for Gene Therapy, Hadassah Hebrew University Medical Center, Jerusalem, Israel; 2 MRI/MRS lab HBRC, Hadassah Hebrew University Medical Center, Jerusalem, Israel; 3 Pediatric Surgery, Hadassah Hebrew University Medical Center, Jerusalem, Israel; 4 Pathology, Hadassah Hebrew University Medical Center, Jerusalem, Israel; 5 Pediatric Hemato–Oncology, Hadassah Hebrew University Medical Center, Jerusalem, Israel; Medical University of South Carolina, United States of America

## Abstract

Neuroblastoma (NB) is the most common extra-cranial pediatric solid tumor with up to 50% of NB patients classified as having high-risk disease with poor long-term survival rates. The poor clinical outcome and aggressiveness of high-risk NB strongly correlates with enhanced angiogenesis, suggesting anti-angiogenic agents as attractive additions to the currently insufficient therapeutics. TL-118, a novel drug combination has been recently developed to inhibit tumor angiogenesis. In the current study, we used the SK-N-BE (2) cell line to generate orthotopic NB tumors in order to study the combinational therapeutic potential of TL-118 with either Gemcitabine (40 mg/kg; IP) or Retinoic acid (40 mg/kg; IP). We show that TL-118 treatment (n = 9) significantly inhibited tumor growth, increased cell apoptosis, reduced proliferation and extended mouse survival. Moreover, the reciprocal effect of TL-118 and Gemcitabine treatment (n = 10) demonstrated improved anti-tumor activity. The synergistic effect of these drugs in combination was more effective than either TL or Gemcitabine alone (n = 9), via significantly reduced cell proliferation (p<0.005), increased apoptosis (p<0.05) and significantly prolonged survival (2-fold; p<0.00001). To conclude, we demonstrate that the novel drug combination TL-118 has the ability to suppress the growth of an aggressive NB tumor. The promising results with TL-118 in this aggressive animal model may imply that this drug combination has therapeutic potential in the clinical setting.

## Introduction

Neuroblastoma (NB), a neoplasm of the sympathetic nervous system, is the most frequent extra-cranial solid tumor in children. It accounts for 7–10% of childhood neoplasms, and 15% of cancer-related deaths in infants [1]. The clinical presentation of NB is highly heterogeneous ranging from spontaneous regression to disseminated aggressive disease [2], with 40–50 percent of them classified as high risk patients. Due to the presence of metastatic disease or MYCN amplification, the five-year event-free survival approaches only 50% despite aggressive treatment [1], [3]. In spite of recent advances using immunotherapy and newly targeted treatments, cure rates for patients with relapsed disease are still extremely low; thus, there is an urgent need for the development of more efficient treatment strategies for NB.

Angiogenesis is a key contributing factor to solid tumor progression, invasion and metastasis [4], [5]. Several studies have shown the association between tumor progression and angiogenesis in NB using *in vivo* and *in vitro* experimental models [6]. Notably, highly vascular NB tumors have been shown to be correlated with MYCN amplification, aggressive disease and poor prognosis [6]–[8]. Current angiogenic inhibitors act to inhibit the angiogenic process either by directly targeting the proliferating endothelial cells or by inhibiting and antagonizing the production of growth factors and downstream effectors involved in the process. A few pre-clinical and early phase clinical trials have been conducted using angiogenesis inhibitors for NB treatment; however, none has yet demonstrated significant large scale results [6]. TL-118, a novel drug combination has been recently developed and optimized to simultaneously inhibit angiogenesis through several independent mechanisms. It is composed of four agents, all reported to have mild to moderate anti-angiogenic effects: (i) Low-dose-high-frequency cyclophosphamide which causes tumor endothelial-cell apoptosis [9]; (ii) The non-steroidal anti-inflammatory drug (NSAID) diclofenac, that targets inflammatory cells, particularly monocytes, and plays a pivotal role in the early stages of angiogenesis [10], [11]; (iii) Sulfasalazine, an NF-kappaB inhibitor [12] that inhibits angiogenesis [13] most likely through the inhibition of vascular smooth muscle cells [14], and (iv) Cimetidine, a histamine H2 receptor blocker that displays anti-angiogenic activity most likely by inhibiting downstream targets of mast cells which are known to play a role in the angiogenic process [15]–[17]. All TL-118 components are approved drugs, available in oral forms, maximizing patient convenience, compliance and safety [18], [19]. Pre-clinical studies have demonstrated that TL-118 significantly inhibited the growth of colorectal liver metastasis [20] and currently the drug is evaluated within two phase II clinical trials for pancreatic cancer and metastatic castration-resistant prostate cancer (NCT00684970; NCT01509911).

The aim of the current study was to assess the therapeutic potential of TL-118 alone and in combination with either retinoic acid (RA) or Gemcitabine (Gem) for the treatment of NB. Currently, RA is a standard treatment for high-risk NB patients following hematopoietic stem cell transplantation (HSCT) [21] and has been shown to significantly improve overall survival [22]. In addition to the retinoids' role as inducers of differentiation and apoptosis [23], several reports have demonstrated their function as angiogenic inhibitors [24]. Retinoids were shown to reduce VEGF secretion production in normal human keratinocytes [25] and to inhibit angiogenesis in thyroid [26] and prostate [27] cancers. Gemcitabine (Gem), an anti-metabolite chemotherapeutic agent, is a cytotoxic drug that directly drives tumor cells to apoptosis. Gem is widely used for the treatment of pancreatic cancer. A few studies have investigated Gem for the treatment of pediatric tumors [28] and anti-tumor activity was observed when Gem was combined with Docetaxel for the treatment of Ewing sarcoma [29], [30]. Currently, the combination of TL-118 with Gem is being assessed in phase II clinical trials for prostate and pancreatic cancer (NCT00684970; NCT01509911).

Noninvasive imaging strategies, and their derived biomarkers of therapeutic efficacy, are attractive techniques since they can facilitate and accelerate drug development [31]. In this study, hemodynamic response imaging (HRI) a functional MRI (fMRI) method that involves hypercapnic and hyperoxic challenges [32] was performed to characterize the therapeutic response of a human NB xenograft model to the novel anti-angiogenic drug combinations of TL-118 [20].

By combining the TL cocktail with either RA or Gem, we aimed to maximize the anti-angiogenic and anti-tumor effects for NB treatment. Our results show that the novel TL-118 drug has the capability to suppress the growth of an aggressive vascular NB tumor. Moreover, the combination of TL-118 with Gem, even at a lower dose, further inhibited tumor growth, suppressed cell proliferation, and significantly increased survival.

## Materials and Methods

### Cell Lines

For NB xenograft generation, three human NB cell lines, at different stages of differentiation were used: the MHH-NB-11 matured NB cell-line [33], the SH-SY5Y poorly differentiated cell-line [33] and the SK-N-BE (2) undifferentiated cell-line [34]. The human MYCN amplified SK-N-BE (2) cell line was kindly provided by Prof. Elizabeth Beierle (Birmingham Alabama, USA; September 2008).The human SH-SY5Y and MHH-NB-11 cell lines were kindly provided by Prof. Isaac Witz (Tel Aviv University, Israel; December 2008). All NB cell lines were routinely grown in RPMI medium (Beit-Haemek, Israel) supplemented with 10% FCS, 1% penicillin-streptomycin and 1% L-glutamine. All cell lines were checked and found to be mycoplasma free.

### Ethics statement

All experiments were performed in accordance with the guidelines and approval of the Institutional Animal Care and Use Committee (IACUC) of the Hebrew University, which holds NIH approval (OPRR-A01-5011).

### In-vivo NB orthotopic model

Human NB cells, (SK-N-BE (2), SH-SY5Y and MHH-NB-11), were orthotopically injected into the left adrenal gland of 6–8week old anesthetized (by intraperitoneal (IP) injection of Ketamine (134 mg/kg) and Xylazine (51 mg/kg)) male NOD-SCID mice (Harlan; Ein-Kerem, Israel). First, a left side high-paracostal approach to the abdomen allowed visualization of the left kidney. A 27-gauge needle was introduced through the left adrenal fat pad and 10^6^ cells/50 _µl_ PBS were inoculated to the adrenal gland. Finally, the skin was closed by surgical sutures. Mice were daily monitored and for tumor follow-up, mice were scanned bi-weekly by MRI. All animal were humanely euthanized when tumors reached the size of 800 mm∧3 as calculated from MRI scans. If mice showed symptoms of weakness or toxicity they received s.c. injection of saline, if their condition was not improved they were humanely euthanized. In all experiments mice were scarified by IP overdose injection of pentobarbital.

Inoculation with MHH-NB-11 cells failed to generate tumors (n = 6) up to day 50. The poorly differentiated SH-SY5Y cells produced orthotopic tumors (n = 9), in 50% of the mice, with an exponential growth kinetics starting 20 days post cell injection. These tumors were morphologically characterized with features resembling rosettes (Figure S1B). The fastest tumor progression was observed with the undifferentiated SK-N-BE (2) cells (n = 19), in 100% of the inoculated mice, demonstrating an aggressive and invasive growth behavior (Figure S1A, C). Therefore, SK-N-BE (2) cells were further used for the evaluation of anti-tumor and antiangiogenic treatments effects.

### Treatments

All treatments were started only after tumor detection by MRI (20±4 days post inoculation). Mice presenting similar tumor size, as detected by MRI, were randomly assigned to the different treatment groups. Treatment combinations were administered daily by IP injection at a volume of 8 ml/kg/day (five days/week) according to the treatment schedules (Table 1).

**Table 1:** Treatment schedule.

	Sunday	Monday	Tuesday	Wednesday	Thursday
TL-118 ^1/4^	TL ^NON-TOX^	TL ^TOX^	TL ^NON-TOX^	TL ^NON-TOX^	TL ^TOX^
TL-118^1/4^ + 13-cis RA (40 mg/kg)	RA + TL ^NON-TOX^	RA + TL ^TOX^	RA + TL ^NON-TOX^	RA + TL ^NON-TOX^	RA + TL ^TOX^
TL-118^1/4^ + Gem (40 mg/kg)	Gem	TL ^TOX^	TL ^NON-TOX^	TL ^NON-TOX^	TL ^TOX^
Gemcitabine (40 mg/kg)	Gem	–	–	–	–

The TL-118 drug combination (Tiltan Pharma Ltd., Jerusalem, Israel) [35] is composed of a low-dose cytotoxic agent (*cyclophosphamide)*, a COX1/2 inhibitor (*diclofenac*), a histamine type 2 (H2) receptor antagonist (*cimetidine*) and an NF-kB inhibitor (*sulfasalazine*). The standard scheduling of TL-118 was divided into two arms – TL-118 ^TOX^ and TL-118 ^NONTOX^ according to previous optimization [20]. An initial experiment was performed with the clinical equivalent dose [20] (TL-118^CLIN^; n = 10). Although TL-118^CLIN^ has been previously shown to have superior anti-tumor activity in a CRLM mouse model [20] without any toxic effects, the highly sensitive immune-deficient mice could not tolerate the TL-118^CLIN^ drug dosage and most of the mice showed adverse toxic side effects (gastric dilatation and weight loss) enforcing us to humanely euthanized them. After additional optimization in naive NOD-SCID mice, we diluted the cytotoxic-TL component to a quarter of the clinical dosage (TL-118^1/4^). The final TL-118^1/4^ component composition is outlined in Table 2. Finally, the reduced treatment formulation- TL-118^1/4^ was administered according to the treatment schedule (Table 1, first row; n = 9).

**Table 2:** TL-118^¼^ drug composition.

	TL^TOX^	TL^NON-TOX^
**Cyclophosphamide** (mg/kg^−1^)	15	
**Diclofenac** (mg/kg^−1^)	7.5	
**Sulfasalazine** (mg/kg^−1^)	125	150
**Cimetidine** (mg/kg^−1^)	15	60

Subsequently, the synergistic effects of RA and Gem with TL-118^1/4^ were assessed. The rational of the different dosing was derived from the pre-existing protocols of pre-clinical studies using these regimens. The combined treatment of TL-118^1/4^ with RA was achieved by the addition of 13-cis RA (40 mg/kg, Sigma Aldrich, Israel) to the daily TL-118^1/4^ cocktail (Table 1, second row; n = 5). Studies that used RA treatment in nude mice used a schedule of 40 mg/kg/day [27], [36]. We presumed that the combination of TL and RA was not going to yield major toxicity; therefore we combined them both at their original dose. The combined treatment of TL-118^1/4^ with Gem was achieved by the addition of 40 mg/kg Gemcitabine (NeoCorp Ag) administered once weekly IP at a volume of 4 ml/kg, followed by daily treatment with TL-118^1/4^ for 4 consecutive days (Table 1, third row; (n = 10)). Dosing of Gemcitabine in pre-clinical studies using nude mice has used a schedule of 40 mg/kg/2–4 days a week [28], [37]. Based on the known toxicity of Gemcitabine and the toxicity of TL-118 we decided to reduce the schedule of Gem to once a week followed by TL-118. In addition, the combination of TL-118 + Gem is currently in phase II clinical studies, at which the treatment scheduling is similar to the schedule we used to treat the mice. For comparison purposes, an additional group of mice was treated once weekly with Gem as a single agent (Table 1, fourth row; n = 9). In each experiment 3–5 untreated mice served as controls for cell viability and tumorogenicity validation. All treatments were continued until tumors reached ethical restrictions (800 mm^∧3^) or mice exhibited evidence of poor health.

### MR Imaging

MRI scans were performed on a horizontal 4.7T Biospec spectrometer (Bruker Medical, Ettlingen, Germany) with a 3.5-cm birdcage coil.

**Anatomical MRI:** Mice were anesthetized with Isoflurane (Nicholas Piramal, India; 2% in a mixture of 30∶70 O_2_:N_2_O) and placed in a supine position. Tumor volume was assessed bi-weekly using T_2_-weighted (T_2_W) fast spin echo images (repetition time = 2,000 ms; echo time = 37 ms; in plane resolution = 117 µm; slice thickness = 1 mm).

**HRI:** Changes in tumor perfusion and vascularity were evaluated by HRI on pentobarbital-anesthetized mice (CTS group, Hod-Hasharon, Israel; 30 mg/kg, IP) when mice reached maximum allowed volume. Images were acquired using T_2_^*^-weighted gradient echo images (repetition time = 147 ms; echo time = 10 ms; field of view = 3.4 cm; in plane resolution = 117 µm; slice thickness = 1 mm; 2 averages; 37 sec/image), under normoxic (air), hypercapnic (95% air + 5% CO_2_) and hyperoxic (95% O_2_ + 5% CO_2_) conditions. The hypercapnic- and hyperoxic-reactivity maps are given as the percentage of change of signal intensity (ΔS) as described previously [32].

### Image Analysis and Statistics

Tumor volume was manually assessed from the T_2_W images using Analyze-7.0 (BIR, Mayo Clinic, Rochester, Minnesota). For each subject, an exponential growth curve was fitted to the tumor volume data points (Matlab software) and exponential coefficients (b-values) for each subject were isolated. The b-values, characterizing tumor growth kinetics were used as a representative value of each subject growth rate. The difference between groups was analyzed by two-sided Mann-Whitney *U* test with Bonferroni correction for small numbers. Survival curves were constructed using the Exact Randomization test and statistical significance was determined by the Log-Rank test (Hintze J 2007, NCSS, UT).

HRI maps were generated as reported previously [32], [38], [39] using IDL (Interactive Data Language of ITT Visual Information Solutions, Boulder, Colorado). Tumor-ROI including the entire lesion and liver and kidney ROI's were defined by analysis of the T_2_W images using the Analyze-7.0 software. Mean ΔS values of these ROIs were calculated by including only pixels with a statistical threshold of *P*<.05 (active pixels), as calculated by the one-sided Mann-Whitney *U* test. The percentage of active pixels was calculated for each gas challenge. All values are expressed as means ± SD. The difference between groups was analyzed by the two-sided Mann-Whitney *U* test. Statistical analyses were performed with the Instat Biostatistics software (GraphPad Software Inc. San Diego, California). A *P*-value of <.05 was considered statistically significant.

### Histology and Immunostaining

Formalin-fixed paraffin-embedded sections from control and treated NB tumors were stained with hematoxylin-eosin (H&E) or subjected to immunohistochemistry (IHC) with specific antibodies. Quantification of necrosis was performed on H&E-stained slides using the Ariol image analysis system (Genetix, San Jose, CA, USA). For apoptosis assessment, TUNEL (Terminal deoxynucleotidyl transferase-mediated deoxyuridine triphosphate nick end-labeling) staining was performed using the ApopTag Peroxidase *In Situ* Apoptosis Detection Kit (Millipore, USA), according to the manufacturer's protocol. Proliferation estimation was performed by Bromodeoxyuridine (BrdU) (GE healthcare Amersham™, UK) injection *in-vivo* (1 ml/100 g, IP) 3 hours prior to mouse sacrifices. Labeled cells were detected using mouse monoclonal anti-BrdU antibody (1∶200, Neomarker). Tumor blood vessels were detected using anti-PECAM-1 antibody (CD31; 1∶50; Biocare Medical, Concord, California). Blood vessel maturation was detected using α-smooth muscle actin (α-SMA) antibody (1∶300; Sigma Chemical Co, St. Louis, MO). All immunostains were evaluated in 10 random high-power microscopic fields (HPF) selected in viable tumor regions only (magnification ×400), and the mean value ±SD of positive cells or vessels was calculated. The difference between groups was analyzed by the two-sided Mann-Whitney *U* test using the Instat Biostatistics software (GraphPad Software Inc., San Diego, California).

## Results

**Figure 1:**
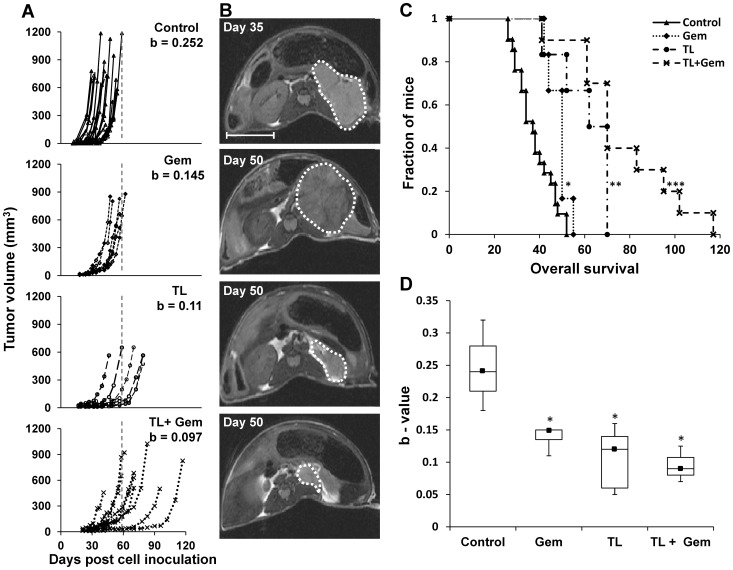
Treatment effect on tumor growth and mouse survival. A. Tumor volume (mm^3^) for each individual mouse, as measured from T_2_W MRI images as a function of days post cell inoculation in control (n = 19), Gemcitabine (Gem; n = 6), TL-118^1/4^ (n = 9) and TL^1/4^+ Gem combination (n = 10) treated mice. The dashed line indicates the maximal survival day of the control-treated mice. The b-values represent the average exponential coefficients of each treatment group. The b-values of all the treated groups (Gem, TL^1/4^ and TL^1/4^+ Gem) were significantly lower compared to control (p<0.0001). B. Representative T_2_W anatomical axial images of Control, Gem, TL-118^1/4^ and TL^1/4^+ Gem treated tumors that were acquired on the indicated days (Bar = 1 cm) C. Kaplan-Meier survival analysis for each of the treated groups (*p<0.05; **P<0.0001; ***p<0.00001 compared to control). D. Box and Whisker plots of mean calculated b-values for each treated group (black square – median; * p<0.0001).

Anatomical T2-weighted MR imaging revealed enlarged adrenal glands from day 12 post cell injection. The detection of initial solid masses above the left kidney was observed approximately 3 to 7 days later. MRI-based assessment of NB tumor progression in this animal model demonstrated exponential tumor growth kinetics with an average survival of 38±12 days (n = 19; Figure 1). The NB tumor location and radiological appearance was consistent with the typical clinical human NB presentation. Histopathological evaluation confirmed the presence of a highly cellular small round blue cell tumor with scant cytoplasm, and virtually no neuropil or Schwannian stroma, consistent with undifferentiated neuroblastoma (NB). The tumors were highly vascular and proliferative with a high mitosis/karyorrhexis index (greater than 4%), indicative of unfavorable histology (Figure S1C).

### Tumor response to TL-118

Although the TL-treatment with the clinical equivalent dose (TL-118^CLIN^) caused severe toxic effects, two out of ten treated mice tolerated the treatment for more than 3 months. In these mice, TL-118^CLIN^ delayed tumor progression and prolonged survival by ∼3-fold (Figure S2A). Moreover, the TL-118^CLIN^ treatment was associated with remarkably reduced tumor vascularization, increased tumor cell apoptosis and suppressed proliferation (Figure S2 B,C).

After elucidating the tolerable TL- dose in NOD-SCID mice, the treatment with TL-118^1/4^ resulted in significant and consistent anti-tumor effects (n = 9). TL-118^1/4^ deferred tumor progression leading to a significantly increased survival (1.5-fold, p<0.0001; Figure 1). The b-values, representing each single tumor growth rate significantly differed between TL-118^1/4^ treated tumors and control tumors (p<0.0001; Figure 1A, D), indicating the reduced growth rate of TL-118^1/4^ treated NB-tumors.

### Tumor response to TL-118^1/4^ + Gem

In the next set of experiments, the synergistic effect of Gem with TL-118^1/4^ (n = 10) was assessed and compared to TL^1/4^ (n = 9) or Gem (n = 6) alone. The treatment with Gem alone showed only mild, yet significant, inhibition of tumor growth. The b-values representing tumor growth rate obtained for Gem-treated tumors were significantly lower than those obtained for control NB tumors (p<0.001), indicating slower tumor growth (Figure 1A). Moreover, this low dose of Gem also significantly prolonged survival by 10 days (p = 0.03; Figure 1C). The TL-118^1/4^+Gem combined treatment demonstrated superior anti-tumor activity compared to each of the single agents, and was significantly more effective in inhibiting tumor growth and increasing survival by 2.5-fold compared to control (p<0.001; Figure 1C). The b-values calculated for TL-118^1/4^+Gem treated-tumors were significantly lower than control, Gem, and TL-118^1/4^ b-values (p = 0.000003, 0.026, 0.003; respectively, Figure 1D). There was no significant difference in body weight loss between mice from the different treatment groups.

### Tumor response to TL-118^1/4^ + RA

Subsequently, the synergistic effect of RA with TL-118^1/4^ (n = 5) was compared to the effect of TL-118^1/4^ alone (n = 5). The TL-118^1/4^ + RA combination did not show beneficial anti-tumor effect compared to TL-118^1/4^ alone (Figure S3). Moreover, tumor kinetics and IHC staining for cell proliferation, apoptosis and blood vessels suggested that the addition of RA even slightly reduced the anti-tumor effect of TL-118^1/4^ (Figure S3).

### Impact of drug combinations on tumor vascularity

**Figure 2:**
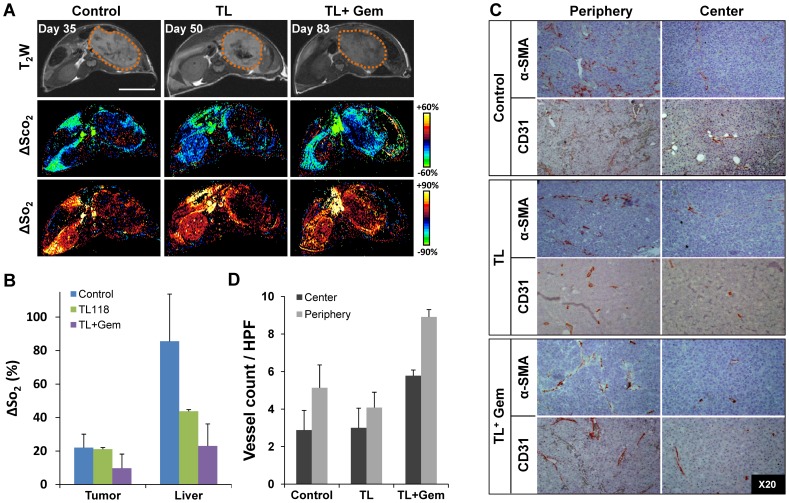
Effects of TL therapy combinations on NB tumor vascularization and perfusion. A. Representative T_2_W fast SE images (top) and the corresponding ΔSco_2_ (middle row) and ΔSo_2_ (bottom) maps of control (left), TL-118^1/4^ (middle column) and TL^1/4^ + Gem (right) treated tumors (Bar = 1 cm). B. Mean ΔSo_2_ values of the tumor and liver-ROI's calculated for control (blue), TL-118^1/4^ (green) and TL^1/4^ + Gem (purple) treated mice. C. Representative histological slides immuno-stained with the smooth muscle marker α-SMA (upper-rows) and with the endothelial cell marker CD31 (lower-rows), of control (top), TL-118^1/4^ (middle) and TL^1/4^ + Gem (bottom) treated tumors. Photographs were taken from the peripheral (left column) and central (right column) regions (original magnification ×20) demonstrating the higher vascularity in tumor periphery. D. Quantification of α-SMA positive vessels/HPF was determined from the tumor center (dark) and peripheral (light) areas for each of the treatment groups. (Mean ± SE; n = 5–7 mice/group).

The HRI-based assessment of the vascular and hemodynamic properties of NB-tumors demonstrated relatively low HRI values compared to the renal and liver HRI-values (Figure 2). Moreover, a heterogeneic response within NB-tumors was observed with higher values in the peripheral areas as opposed to tumor center (Figure 2A). Similarly, CD31 and α-SMA staining confirmed the relatively higher vessel-count in the tumor periphery relative to tumor center (Figure 2C, D). Tumors-HRI values of all treated NB-tumors showed only a slight decrease in HRI values compared to control tumors (Figure 2B). Indeed, when analyzing the corresponding histological specimens, all tumors showed comparable levels of vascularization and no significant difference was observed between treatment groups (Figure 2C). In contrast, HRI results demonstrated a significant decrease in liver-HRI values of all of the treated mice compared to control liver-HRI values (Figure 2B). This observation resembled a phenomenon that was previously proven to be associated with the reduced liver perfusion due to TL-118 therapy in a murine model of colorectal liver metastasis [20].

### Cellular evaluation– treatment effect on tumor cells

**Figure 3:**
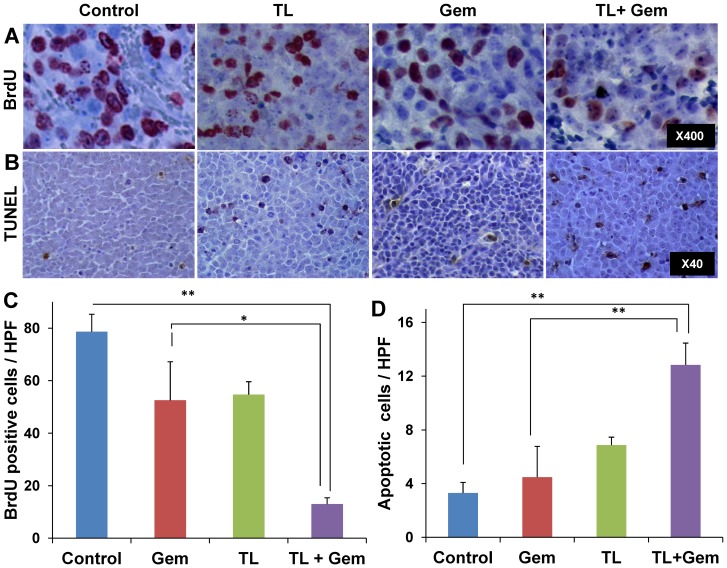
Effects of the different therapies on NB tumor proliferation and apoptosis. Representative histological sections of control (1^st^ column), TL-118^1/4^ (2^nd^ column), Gem (3^rd^ column) and TL^1/4^ + Gem (4^th^ column) treated mice. Slides were immuno-stained for proliferation (BrdU) (A) and apoptosis (TUNEL) (B); Quantification of BrdU positive (C) and TUNEL positive (D) cells/HPF analyzed from 10 randomly selected HPF/tumor ; n = 3–6 mice/group. Original magnification is indicated on the right image of each row. * p = 0.01, ** p<0.005. The histological sections were taken at the end of the experiments when the tumor load/mouse reached ethical limits.

Histopathological analysis of control NB-tumors revealed high cell proliferation and a low percentage of apoptotic cells (Figure 3). Both TL-118^1/4^ and Gem treatments, when given independently, had moderate effects on cell proliferation and apoptosis. In contrast, the combined TL-Gem treatment significantly inhibited cell proliferation (Figure 3A, C; 80% reduction, *p*<0.005), and induced a 4-fold elevation in the number of apoptotic cells compared to control (Figure 3B,C; p<0.005). Computerized analysis of the histological sections demonstrated equivalent necrotic area (6–8%) ratio in all groups.

## Discussion

Fifty to sixty percent of patients with high-risk NB eventually relapse with aggressive disease, resistant to any known treatment strategy [1], [3]. In this report, we present the outcome of a new antiangiogenic combination therapy in an animal model. Our results suggest that this combination may be effective for this group of extremely high-risk patients.

The poor clinical outcome and aggressive tumor phenotype of high-risk NB correlates with enhanced tumor angiogenesis [6], [8], [40]–[42], suggesting both anti-angiogenic and antivascular agents as attractive additions to the currently insufficient therapeutics. Recently, several anti-angiogenic agents have been evaluated for NB treatment in a few clinical trials, but have failed to induce enduring clinical responses [6]. In the present study, we used the MYCN amplified SK-N-BE (2) undifferentiated NB cells to generate aggressive orthotopic tumors, in order to study the therapeutic potential of the novel anti-angiogenic drug combination, TL-118 [20].

The TL-118 cocktail offers a multi-faceted approach to interfering with the complex angiogenic process. The combination acts as an angiogenesis inhibitor by targeting endothelial and pro-inflammatory immune cells which have a preeminent role in the development and maintenance of blood vessels (Tiltan Pharma, Ltd, 2008, 2012). Preliminary results with TL-118^CLIN^ demonstrated remarkable inhibition of tumor growth leading to a ∼3-fold increased survival compared to untreated tumors, with pronounced pro-apoptotic, anti-proliferative and anti-angiogenic effects. However, the severe toxicity in the immune-compromised mice forced us to reduce the dose of the TL-118's cytotoxic agent to one quarter (Table 2) for the rest of the study.

Morphological normalization of tumor vasculature is a phenomenon described during treatment with anti-angiogenic agents [43]. In this process, tumor vessels are characterized as having a more “normal”, organized structure which results in more efficient tumor blood flow and hence, drug delivery [44]. Therefore, co-administration of anti-angiogenic drugs with cytotoxic agents enhances their direct anti-tumor effects. Indeed, the combination of anti-angiogenic treatment with chemotherapy has shown to be more effective for cancer treatment [45]. In fact, the combination of TL-118 + Gem is currently being assessed in phase II clinical trials for the treatment of prostate and pancreatic cancers. Based on the positive preliminary clinical findings, in our experimental setup, we combined TL-118^1/4^ with either RA or Gem, aiming to maximize the anti-angiogenic and anti-tumor effects. Treatment with TL-118^1/4^ alone resulted in significant and consistent anti-tumor effects; TL^1/4^-treatment significantly reduced tumor growth rate, increased cell apoptosis, inhibited proliferation and extended survival by 1.5-fold. Moreover, the reciprocal effect of TL-118^1/4^ and Gem combined treatment demonstrated potent anti-tumor activity. Consistent with a synergistic effect, this combination was more effective than either TL or Gem alone, by significantly reducing cell proliferation, inducing apoptosis and significantly prolonging mouse survival.

In contrast, the combination of TL^1/4^ with RA did not show any beneficial anti-tumor effects compared to TL-118^1/4^ alone. Retinoids are signaling molecules that are known to be involved in proliferation, differentiation and apoptosis [23]. Non genomic actions of RA on NB cells is mediated by the retinoid acid receptor (RAR), which results in the activation of PI3K and MAPK signaling pathways [46]. In addition, it has been shown that RA induces COX-2 and prostaglandin E2 synthesis in human NB SH-SY5Y cells, through the RAR activation of ERK1/2 [47], hence contributing to cell differentiation. One explanation that could account for the lack of a beneficial effect of the RA and TL-118 combination is their potential antagonistic activity. While RA induces COX-2 synthesis, one of TL's components- diclofenac inhibits COX-2 [11]. Besides, the lack of beneficial response in this combined treatment group may be a result of RA dose.

NB has the tendency to invade surrounding tissues and blood vessel walls, making surgical dissection difficult and sometimes dangerous. By using MRI, we were able to utilize an orthotopic NB mouse model which closely resembles the clinical location and presentation of the human disease in order to evaluate and improve the efficiency of new drug combinations non-invasively. Moreover, MRI usage enabled us to initiate the treatments only on the day of tumor detection, thus mimicking the tumors at a minimal residual disease (MRD) state.

In a previous study, TL-118^CLIN^ was well tolerated and showed a significant anti-angiogenic effect on colorectal liver metastasis [20]. In this study, while tumor perfusion and vascularization assessment revealed that only TL^CLIN^ caused a detectable anti-angiogenic effect, tumors from all other treatment groups were highly vascular with no significant difference between them. In accordance, TL^CLIN^ treatment has demonstrated encouraging preliminary evidence of reducing the size of an established tumor with effective anti-tumor and anti-angiogenic effects. The failure to induce a sufficient anti-angiogenic response in this study probably resulted from the need to reduce TL's cytotoxic component so that it can be tolerated by the highly sensitive immune-deficient mice. In order to clarify this point, we expect that by using non immune compromised animal models, such as the TH-MYCN NB model [48], it will be possible to evaluate TL-118^CLIN^ dose potentially for NB therapy.

To conclude, this study shows that the novel drug combination TL-118 has the ability to suppress the growth of an aggressive NB tumor in an animal model and may be a candidate for NB therapy. In addition to TL's tumor inhibitory effects, we showed its synergistic activity when combined with Gemcitabine. Moreover, our findings reinforces the possible use of Gem for NB treatment as has been demonstrated by Ogawa *et al.* [28] and as is currently evaluated in a phase II clinical trial [49]. By blocking multiple angiogenic pathways together with Gem-cytotoxicity, the therapeutic capacity rises and a stronger antitumor effect is achieved. The promising results with the reduced dose of TL-118 in this aggressive animal model may imply that this drug combination has a therapeutic potential at its full dosage in the clinical setting.

## Supporting Information

Figure S1 **Tumor growth kinetics of the different NB cell lines and their clinical presentation.** NB cells (10^6^) were orthotopically injected to the adrenal glands of NOD/SCID mice and tumor progression was followed bi-weekly by MRI. Mean tumor growth kinetics (left) and a representative histological slide stained with H&E (right) of the undifferentiated SK-N-BE(2) cell line (A) and of the poorly differentiated SH-SY5Y cell line (B). Note the rosette-like appearance of SH-SY5Y tumors. Original magnification ×40 (C). Representative anatomical T_2_W coronal (top) and axial (middle) images of SK-N-BE(2) tumor bearing mouse (Bar = 1 cm). Enlarged box on axial image illustrates NB tumor encapsulating a large blood vessel and the corresponding H&E slide of this tumor (bottom) showing the encapsulated vessel (arrow heads).
(TIF)

Figure S2 **The effects of TL-118 clinical equivalent dose on NB tumor growth and vascularization.** TL-118^CLIN^ considerably suppressed NB tumor growth leading to a 3-fold increased survival in 2 mice; unfortunately, the rest (n = 8) suffered from severe toxic effects. (A) Tumor growth kinetics of control (solid line; n = 19) and two individual TL-118^CLIN^ treated mice (dashed lines). (B) Representative histological sections of control (left column) and TL-118^CLIN^ (right column) stained with H&E (1^st^ row), CD 31 (2^nd^ row), BrdU (3^rd^ row) and TUNEL (4^th^ row). TL-118^CLIN^ treated tumors were smaller with less blood vessels compared to control. Moreover, TL-118^CLIN^ significantly reduced cell proliferation and increased apoptosis. (C) Quantification of CD31 positive vessels, BrdU positive cells and TUNEL positive cell immunostaining.
(TIF)

Figure S3 **The effects of TL-118^1/4^ + RA combination.** RA addition to TL-118 had no beneficial therapeutic effect on NB tumors. (A) Mean tumor growth kinetics of control (blue line; n = 5), TL-118^1/4^ (green line; n = 5) and TL-118^1/4^ +RA (red line; n = 3) treated mice. (B) Representative histological sections of control (Top) and TL-118^1/4^ + RA (Bottom) stained with TUNEL (left) for apoptosis and KI67 (right) for proliferation. (C) Quantification of the TUNEL positive cells demonstrated the improved killing effect of TL-118^1/4^ alone.
(TIF)
