# Supplementary figures and images for: TL-118 and Gemcitabine Drug Combination Display Therapeutic Efficacy in a MYCN Amplified Orthotopic Neuroblastoma Murine Model – Evaluation by MRI
Source: PLoS One. 2014 Mar 6;9(3):e90224. doi: 10.1371/journal.pone.0090224 (PMC3946152; doi:10.1371/journal.pone.0090224)

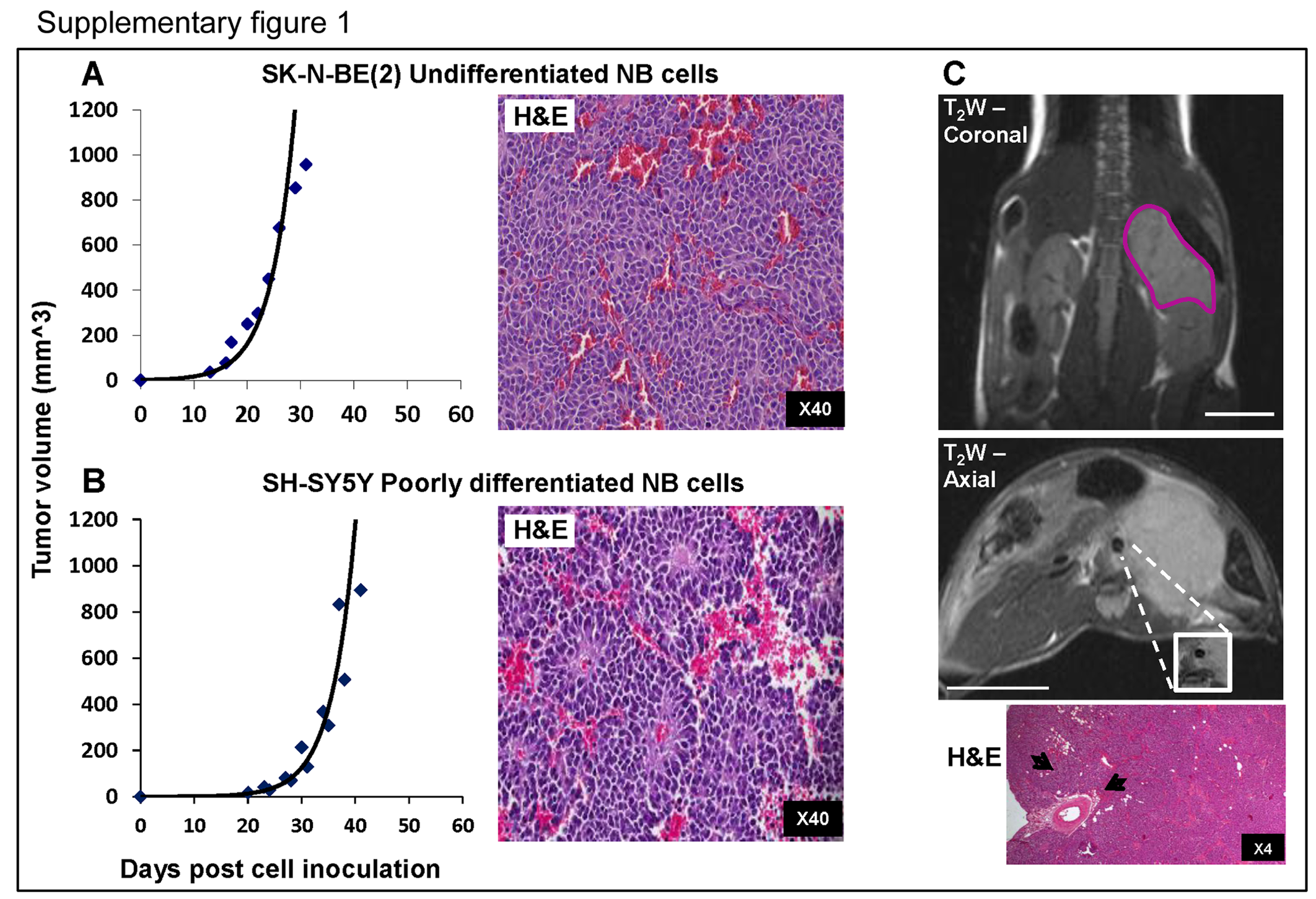

Supplement: Figure S1 — Tumor growth kinetics of the different NB cell lines and their clinical presentation. NB cells (106) were orthotopically injected to the adrenal glands of NOD/SCID mice and tumor progression was followed bi-weekly by MRI. Mean tumor growth kinetics (left) and a representative histological slide stained with H&E (right) of the undifferentiated SK-N-BE(2) cell line (A) and of the poorly differentiated SH-SY5Y cell line (B). Note the rosette-like appearance of SH-SY5Y tumors. Original magnification ×40 (C). Representative anatomical T2W coronal (top) and axial (middle) images of SK-N-BE(2) tumor bearing mouse (Bar = 1 cm). Enlarged box on axial image illustrates NB tumor encapsulating a large blood vessel and the corresponding H&E slide of this tumor (bottom) showing the encapsulated vessel (arrow heads). (TIF) [file pone.0090224.s001.tif]

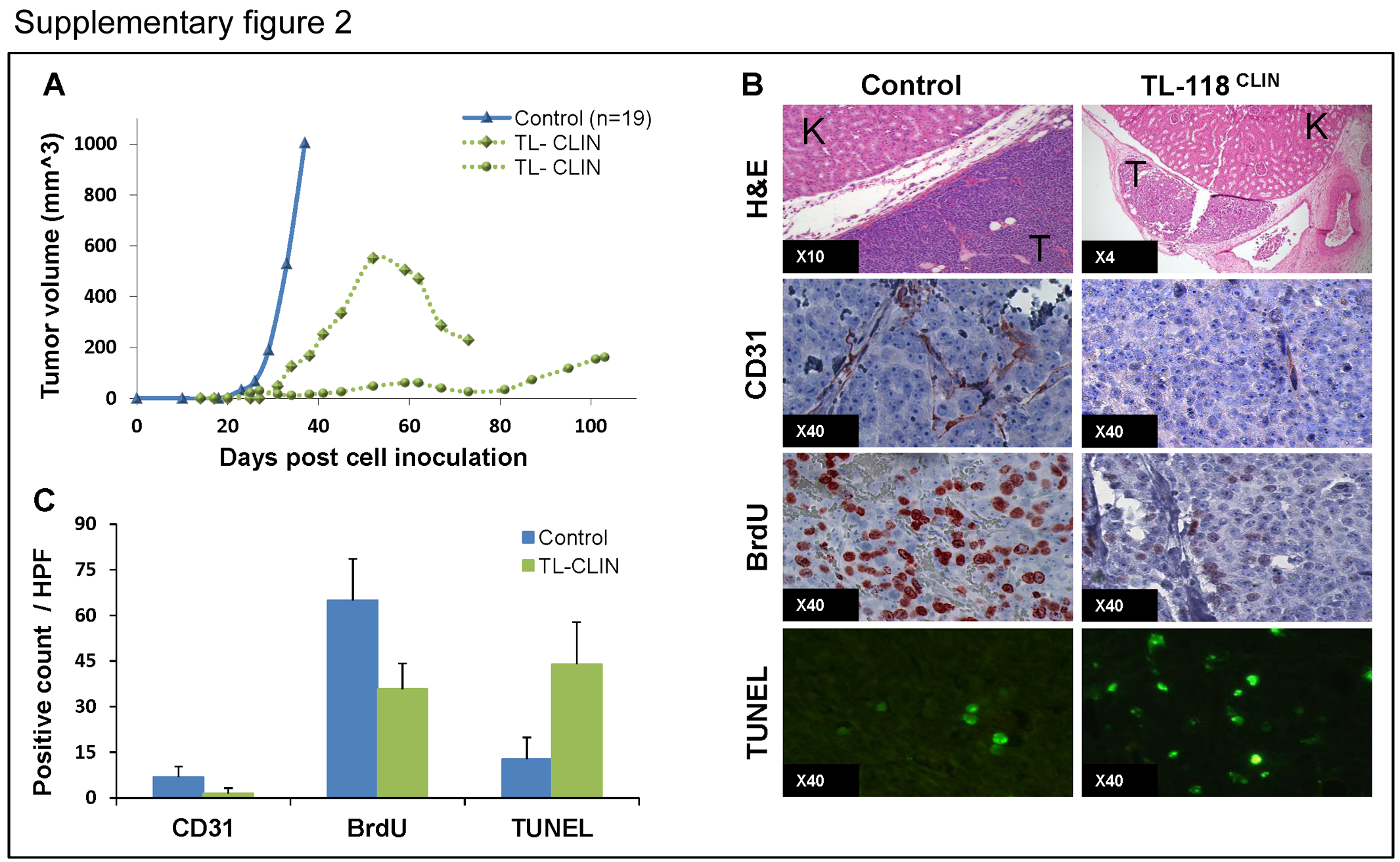

Supplement: Figure S2 — The effects of TL-118 clinical equivalent dose on NB tumor growth and vascularization. TL-118CLIN considerably suppressed NB tumor growth leading to a 3-fold increased survival in 2 mice; unfortunately, the rest (n = 8) suffered from severe toxic effects. (A) Tumor growth kinetics of control (solid line; n = 19) and two individual TL-118CLIN treated mice (dashed lines). (B) Representative histological sections of control (left column) and TL-118CLIN (right column) stained with H&E (1st row), CD 31 (2nd row), BrdU (3rd row) and TUNEL (4th row). TL-118CLIN treated tumors were smaller with less blood vessels compared to control. Moreover, TL-118CLIN significantly reduced cell proliferation and increased apoptosis. (C) Quantification of CD31 positive vessels, BrdU positive cells and TUNEL positive cell immunostaining. (TIF) [file pone.0090224.s002.tif]

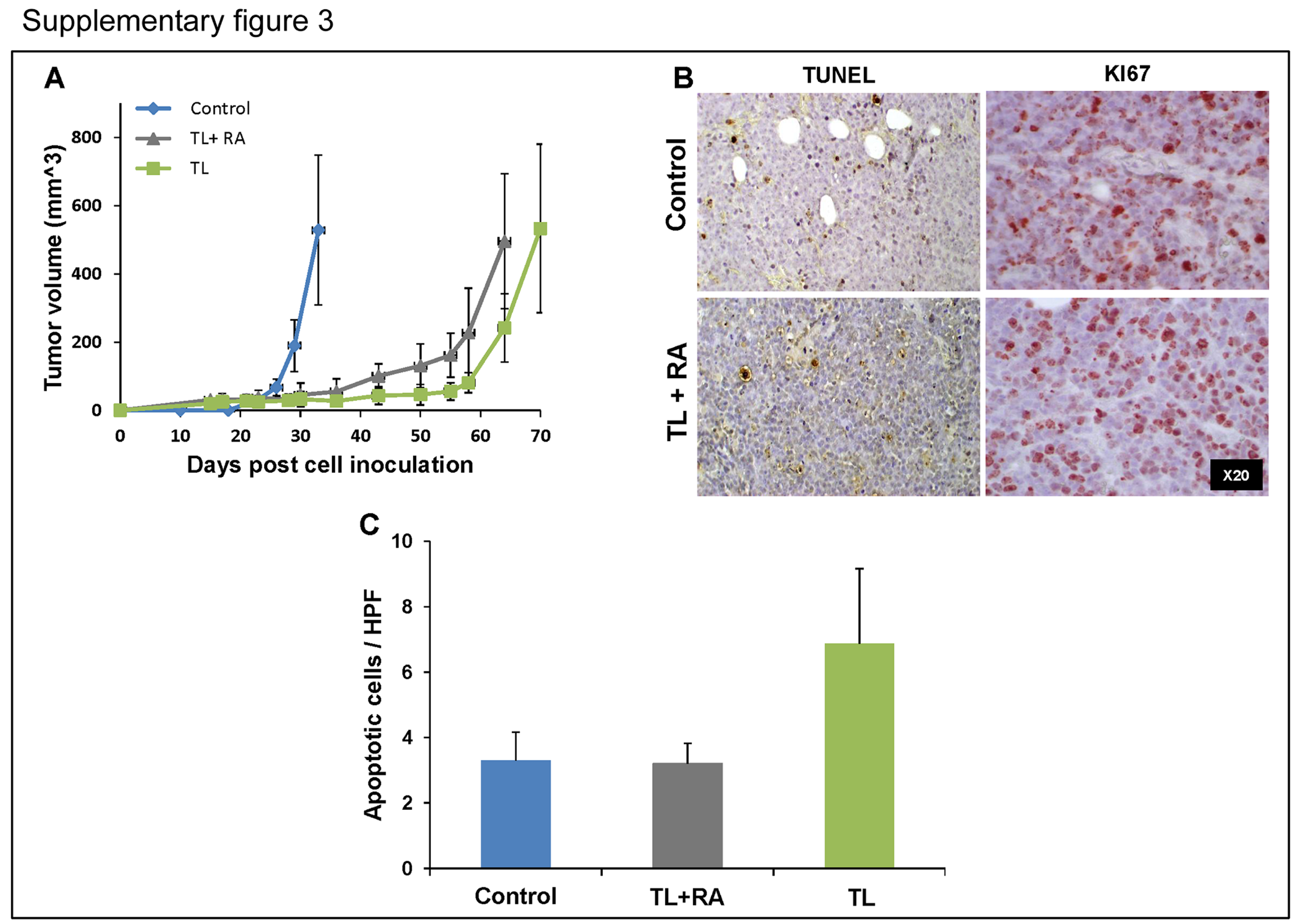

Supplement: Figure S3 — The effects of TL-1181/4 + RA combination. RA addition to TL-118 had no beneficial therapeutic effect on NB tumors. (A) Mean tumor growth kinetics of control (blue line; n = 5), TL-1181/4 (green line; n = 5) and TL-1181/4 +RA (red line; n = 3) treated mice. (B) Representative histological sections of control (Top) and TL-1181/4 + RA (Bottom) stained with TUNEL (left) for apoptosis and KI67 (right) for proliferation. (C) Quantification of the TUNEL positive cells demonstrated the improved killing effect of TL-1181/4 alone. (TIF) [file pone.0090224.s003.tif]
